# *Myrtus communis* L. Essential Oil Exhibits Antiviral Activity against Coronaviruses

**DOI:** 10.3390/ph17091189

**Published:** 2024-09-10

**Authors:** Dar-Yin Li, Matthew G. Donadu, Taylor Shue, Georgios Dangas, Antonis Athanasiadis, Shuiyun Lan, Xin Wen, Basem Battah, Stefania Zanetti, Vittorio Mazzarello, Stefan G. Sarafianos, Marco Ferrari, Eleftherios Michailidis

**Affiliations:** 1 Laboratory of Biochemical Pharmacology, Department of Pediatrics, Center for ViroScience and Cure, Emory University School of Medicine, and Children’s Healthcare of Atlanta, 1750 Haygood Drive, Atlanta, GA 30322, USA; darli@utmb.edu (D.-Y.L.); taylor.shue@emory.edu (T.S.); georgios.dangas@emory.edu (G.D.); antonis.athanasiadis@emory.edu (A.A.); shuiyun.lan@emory.edu (S.L.); xin.wen@emory.edu (X.W.); stefanos.sarafianos@emory.edu (S.G.S.); 2 Scuola di Specializzazione in Farmacia Ospedaliera, Department of Medicine, Surgery and Pharmacy, University of Sassari, 07100 Sassari, Italy; mdonadu@uniss.it; 3 Hospital Pharmacy, Giovanni Paolo II Hospital, ASL Gallura, 07026 Olbia, Italy; 4 Department of Biochemistry and Microbiology, Antioch Syrian Private University, M5, Damascus 22734, Syria; basem.battah.sc@hotmail.com; 5 Department of Biomedical Sciences, University of Sassari, 07100 Sassari, Italy; stefania.zanett_2020@libero.it (S.Z.); vmazza@uniss.it (V.M.); 6 Institute for Maternal and Child Health, IRCCS Burlo Garofolo, Via dell’Istria, 65, 34137 Trieste, Italy; dr.marcoferrari@gmail.com

**Keywords:** Myrtus, antiviral drugs, HCoV-229E, HCoV-OC43, SARS-CoV-2, essential oil, natural products, therapeutics, host defense

## Abstract

Human coronaviruses are a continuous threat to the human population and have limited antiviral treatments, and the recent COVID-19 pandemic sparked interest in finding new antiviral strategies, such as natural products, to combat emerging coronaviruses. Rapid efforts in the scientific community to identify effective antiviral agents for coronaviruses remain a focus to minimize mortalities and global setbacks. In this study, an essential oil derived from *Myrtus communis* L. (MEO) is effective against HCoV-229E and HCoV-OC43 virus infections in comparison to two FDA-approved drugs, Remdesivir and Nirmatrelvir. Gas-chromatography and mass spectrometry were used to identify the chemical composition of MEO. Slight antioxidant activity was observed in MEO, indicating a role in oxidative stress. A dose–response curve measuring the EC_50_ indicates a high potency against HCoV-229E and HCoV-OC43 virus infections on Huh7.5 cells with low cytotoxicity using a PrestoBlue cell viability assay. Our findings demonstrate that MEO exhibits potent antiviral activity against HCoV-229E and HCoV-OC43 on Huh7.5 cells within a low-cytotoxicity range, but not on SARS-CoV-2. Artificial bacterial chromosome plasmids that expressed SARS-CoV-2 used for replicon—to determine viral replication and viral assembly/egress on HEK293T/17 cells—and virus-like particles on Huh7.5-AT cells—to determine viral entry and assembly/egress—showed no antiviral activity with MEO in comparison to Remdesivir. This study reveals the potential effectiveness of MEO as an alternative natural remedy to treat human coronaviruses and a potential antiviral agent for future coronavirus infections.

## 1. Introduction

The recent COVID-19 pandemic has resulted in the deaths of 6.9 million people as of November 2023 and has led to unprecedented changes in daily functions [1]. Human coronaviruses, such as HCoV-229E and HCoV-OC43, are positive-sense, single-stranded RNA viruses that not only affect the respiratory system with mild to severe symptoms, but also affect the nervous system [2]. With no current approved antiviral drug for HCoV-229E and HCoV-OC43 viruses which cause upper respiratory tract infections, examining various antiviral options allows us to lessen the severity of infection and reduce the risk of hospitalization [3]. SARS-CoV-2, while similar to HCoV-229E and HCoV-OC43, has distinct features in that SARS-CoV-2 predominantly affects the lower respiratory tract, but recent variants have been shown to evolve upper respiratory traits [4]. Furthermore, viral entry for SARS-CoV-2 utilizes ACE2 receptors on host cells and TMPRSS2 for spike protein priming, while HCoV-229E and HCoV-OC43 bind to human aminopeptidase N and 9-O-acetylated-sialic acid, respectively [5,6,7]. Moreover, studies on common cold coronaviruses provide insights into antiviral strategies for future highly pathogenic coronaviruses similar to SARS-CoV-2.

Expanding on the topic of aromatic and medicinal plants, particularly focusing on their antiviral activity, we delve into the potential for these plants to combat viral infections. Aromatic and medicinal plants, like *Myrtus communis* L., are not only valued for their traditional uses and economic impact across industries such as agriculture, perfumery, cosmetics, and pharmacy, but also for their potential in addressing viral diseases. These plants contain a rich composition of bioactive compounds like alkaloids, flavonoids, phenols, tannins, vitamins, and essential oils that exhibit significant antiviral properties [8,9,10,11].

For instance, the essential oil derived from Myrtus (MEO), well-known for its application in treating respiratory and gastrointestinal ailments, shows promising antiviral effects. Studies have indicated that MEO can inhibit the replication of certain viruses, making it a potential candidate for the development of antiviral drugs, and it is particularly relevant in the context of viral outbreaks where conventional medications might be limited or ineffective [12].

The antiviral activity of these plants can be attributed to their ability to interfere with various stages of the viral life cycle, from entry to replication and release. Some compounds found in these plants can disrupt the viral envelope or interfere with viral proteins, thus impeding the virus’s ability to infect host cells. Additionally, the anti-inflammatory and immunomodulatory properties of these plants can enhance the body’s natural defense mechanisms against viral infections, as Myrtus can inhibit human herpes virus through antiviral screening [13,14].

The previous literature also indicates that phillyrin, an ingredient derived from the plant *Forsythia suspensa*, possesses antiviral activity against HCoV-229E in Huh7 cells, demonstrating that natural products can inhibit coronavirus proliferation [15]. Furthermore, the phytochemical, lycorine, from *Lycoris squamigera*, can inhibit SARS-CoV-2 and HCoV-OC43, showing effectiveness against viruses in the genus *Betacoronavirus* [16]. Exploring antiviral properties in aromatic and medicinal plants is part of a broader field of research seeking natural solutions to contemporary health challenges. By studying and exploiting the properties of plants such as Myrtus, scientists aim to develop new and effective antiviral agents that can complement or offer alternatives to synthetic drugs. This research not only highlights the importance of these plants in traditional medicine, but also opens new avenues for their application in modern pharmacology, especially in the fight against viruses like coronaviruses.

Thus, this study was conducted to examine whether MEO exhibits antiviral activity using dose–response curves against HCoV-229E and HCoV-OC43 infection on Human hepatoma-derived cells (Huh7.5) in comparison to Remdesivir and Nirmatrelvir. In addition, SARS-CoV-2 subgenomic replicon and virus-like particle (VLP) systems were used to investigate if MEO possesses antiviral activity against SARS-CoV-2 and whether MEO affects replication or viral entry/egress. We measured the chemical composition of the essential oil from Myrtus through gas chromatography and mass spectrometry and quantified the antioxidant activity of the essential oil to determine its health benefits.

## 2. Results

### 2.1. Chemical Compositions of MEO

The aerial parts of *Myrtus communis* L. from Tempio Pausania, when steam distilled, produced colorless essential oil derived from Myrtus with yields between 0.32% and 0.52% (*v*/*w*) based on dry weight. The MEO was analyzed using GC-MS for qualitative and (GC-FID) with n-dodecane as an internal standard for quantitative assessment. This process identified 31 components, accounting for 92.2–95.6% of the total sample. Notably, α-pinene with a retention time of approximately 13 min was predominant in the leucocarpa variety at concentrations of 270 mg/mL and 212 mg/mL in cultivated and wild plants, respectively (Table 1). Consistent with the literature, α-pinene is often the main compound in *M. communis* leaf essential oils (EO) [17,18,19,20,21]. However, EOs from some M. communis samples in Morocco, Greece, and Spain [10,11,12,13] are distinguished by the presence of Myrtenyl acetate [19,22,23,24]. Thus, the major chemical compositions in MEO are terpenoid compounds (1,8-cineole, α-pinene, Myrtenyl acetate, limonene, linalool, and α-terpinolene), as these compounds are found in leaves in most geographical regions [25].

In this study, linalool was identified as a key component in wild plants with a concentration of 7.26 mg/mL in MEO. This aligns with a previous study’s classification of MEO in Algeria, where two groups were distinguished as follows: “Group I” with high α-pinene and 1,8-cineole levels (similar to cultivated plants in this study), and “Group II” with higher amounts of linalool and linalyl acetate [17]. Although MEO in Algeria have no correlation between environmental conditions and chemical composition, another study observed that *M. communis* grown in partial shade and siliceous soil had a volatile profile rich in linalool, linalyl acetate, and oxygenated monoterpene and sesquiterpene compounds, resembling the wild samples in this study [17,20].

### 2.2. Antioxidant Activity

The total antioxidant activity of MEO was evaluated using the DPPH radical dot test. In vitro, an analysis of MEO indicated a slight level of antioxidant activity (Table 2). These findings are in line with earlier research on MEOs from Iranian and Moroccan sources of *M. communis* [22,26]. The DPPH assay, a widely accepted method for assessing antioxidant properties, measures the ability of MEO to scavenge free radicals, thus providing insights into their potential therapeutic applications. The slight antioxidant activity observed suggests that MEO could have beneficial effects, particularly in contexts where oxidative stress plays a role. This may be attributed to the hydroalcoholic extracts of MEO which contain high amounts of polyphenols, galloyl-glucosides, ellagitannins, galloyl–quinic acids, and flavonol glycosides that are well known for their antioxidant activities [27]. Although our MEO did not exhibit those chemical compositions and was extracted from a different region of Sardinia, our MEO processed terpinenes, which contribute to the slight antioxidant activity.

In a recent study, coronaviruses, such as a SARS-CoV-2 infection of the host cells, resulted in heightened reactive oxygen species and weakened antioxidant host responses; thus, high levels of antioxidant activity compounds like curcuminoids increase *NRF2* gene expression and inhibit SARS-CoV-2 infection [28]. This aligns with the growing body of research underscoring the importance of natural antioxidants in health and disease prevention.

### 2.3. Antiviral Activity against HCoV-229E Coronavirus

**Figure 1:**
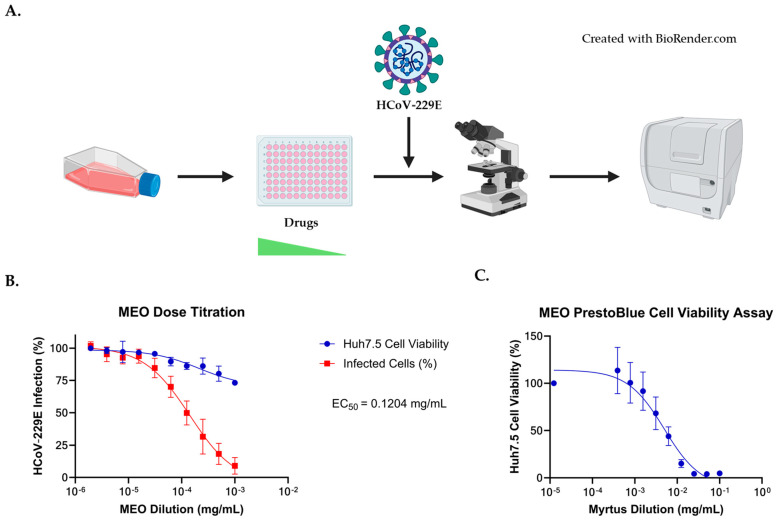
MEO inhibits HCoV-229E infection. (**A**) Experimental design of MEO experiment for dose–response curve. Huh7.5 cells were seeded in two 96-well plates with Remdesivir and Nirmatrelvir as antiviral controls. The experiment was performed in triplicate, and the starting concentration for MEO was 1:1000 (0.8469 mg/mL) and received a 1:2 serial dilution. The starting concentrations for Remdesivir and Nirmatrelvir were 250 nM and 25 µM, respectively, and were diluted with a 1:2 serial dilution. After HCoV-229E was added to the plates an hour after drug treatment, cells were fixed with 4% PFA one day post-infection. Immunofluorescent staining (IF) was performed to visualize infected cells using Cytation 7. (**B**) Dose–response curve showing that MEO has antiviral activity against HCoV-229E with EC_50_ = 0.1204 mg/mL MEO concentration starting at 1:1000 (0.8469 mg/mL) with a 1:2 serial dilution and infected with 1:10 HCoV-229E virus. (**C**) Cytotoxicity assay measuring cell viability in MEO-treated Huh7.5 cells. A 1:10 (84.69 mg/mL) starting concentration for MEO was used with a 1:2 serial dilution. Huh7.5 cells treated with MEO were normalized to the untreated Huh7.5 cells.

To determine the potential antiviral effects of MEO against in vitro infection of HCoV-229E, Huh7.5 cells were treated with Remdesivir, Nirmatrelvir, and MEO using a 1:2 serial dilution in a 96-well plate format (Figure 1A). Remdesivir and Nirmatrelvir, which are effective antivirals against the coronavirus family, acted as positive controls to compare MEO potency against HCoV-229E virus. Huh7.5 cells were infected with HCoV-229E after one hour of drug pre-treatment. Immunostaining two days post-infection was used to generate a dose–response curve for each drug. The dose–response curve showed an EC_50_ of 0.1204 mg/mL in infected media (DMEM with 10% FBS and 1% NEAA) of MEO using Cytation 7 (Figure 1B). The starting concentration for MEO was 1:80 (10.5863 mg/mL), but MEO treatment was toxic at concentrations above 1:160 (5.2931 mg/mL); thus, points that were cytotoxic were removed from EC_50_ calculations for accuracy (Figure 1B). A PrestoBlue cell viability assay confirmed that Myrtus concentrations above 1:160 (5.2931 mg/mL) were cytotoxic (Figure 1C). The CC_50_ value for MEO on Huh7.5 cells was 4.197 mg/mL (Table 3).

**Figure 2:**
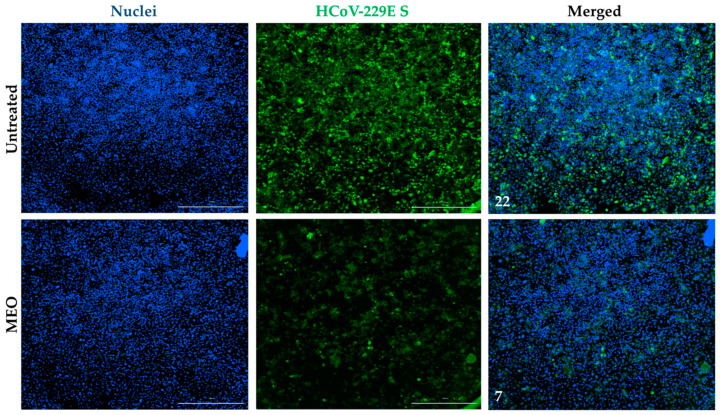
Immunofluorescent staining for HCoV-229E Spike protein. Huh7.5 cells without MEO treatment showed a mean fluorescence intensity of 22 with HCoV-229E. At a 1:2000 (0.42345 mg/mL) Myrtus concentration, the HCoV-229E viral infection has a mean fluorescence intensity of 7. Nuclei were stained with Hoechst, and HCoV-229E was stained with HCoV-229E spike protein and Alexa Fluor 488, labeled goat anti-mouse secondary antibody, and was imaged at a 1000 µm scale.

In parallel to high-content, high-throughput imaging using Cytation 7, a 1:2000 (0.42345 mg/mL) concentration of MEO in a 96-well plate was specifically captured to compare the infection rate of HCoV-229E with an untreated well. In comparison with the HCoV-229E-captured image of the untreated well, a 0.42345 mg/mL concentration of Myrtus has a lower percentage of infection with a mean fluorescence intensity (MFI) of 7 while the untreated well has an MFI of 22 using ImageJ (Figure 2). With a small dose of Myrtus essential oil in the drug titration assay, HCoV-229E infection was inhibited while the Huh7.5 cells were still viable with low cytotoxicity. This is supported by the MFI of the merged immunofluorescence images, with MFI much greater in the infected well without MEO treatment than the MEO-treated well. Thus, the phenotype of the wells validates the dose–response curve assay that HCoV-229E infection is inhibited against Huh7.5 cells with MEO treatment within the non-cytotoxicity range (Figure 1 and Figure 2).

### 2.4. Comparison of EC_50_ and CC_50_ with HCoV-229E Infection for MEO, Remdesivir, and Nirmatrelvir

MEO exhibited promising inhibition against HCoV-229E infection with an EC_50_ of about 0.1204 mg/mL and appears to have low cytotoxicity with a CC_50_ of 4.197 mg/mL (Table 3). MEO cytotoxicity is about 35-fold higher than EC_50_; thus, the essential oil exhibits an antiviral effect without killing the cells. Because MEO is derived from various chemical compositions, the exact concentration was not able to be extrapolated and is measured in relative dilution instead. Remdesivir and Nirmatrelvir were run in parallel with MEO as control compounds, with EC_50_ demonstrating high potency against HCoV-229E with 0.003 nM and 1.243 nM, respectively. Similarly, Remdesivir and Nirmatrelvir both exhibited low cytotoxicity (Table 3).

### 2.5. Antiviral Activity against HCoV-OC43 Coronavirus

**Figure 3:**
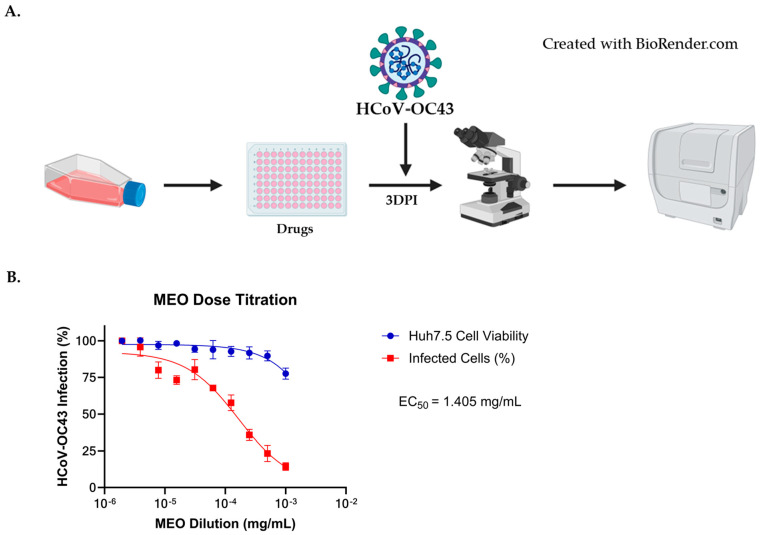
MEO inhibits HCoV-OC43 infection. (**A**) Experimental layout of MEO with HCoV-OC43 infection to determine EC_50_. Two collagen-coated 96-wells plates were seeded with Huh7.5 cells to evaluate the dose–response curve of Remdesivir, Nirmatrelvir, and MEO. Each drug was conducted in triplicates with the starting concentration for MEO to be 1:1000 (0.8469 mg/mL) and serially diluted 1:2. The starting concentrations for Remdesivir and Nirmatrelvir were 7 µM and 25 µM, respectively, and were diluted with a 1:2 serial dilution. An hour after drug treatment, HCoV-OC43 was added, and cells were fixed with 4% PFA at 3 days post-infection. Immunofluorescent staining (IF) was performed to visualize infected cells using the Cytation 7. (**B**) A dose–response curve showed that MEO has antiviral activity against HCoV-OC43 with EC_50_ = 1.405 mg/mL MEO concentration starting at a concentration of 1:1000 (0.8469 mg/mL) with a 1:2 serial dilution and infected with 1:20 HCoV-OC43E virus.

A similar methodology from the HCoV-229E assay was used to determine MEO antiviral activity against HCoV-OC43. Remdesivir and Nirmatrelvir were also used to act as positive controls to determine the efficacy of MEO against HCoV-OC43 in Huh7.5 cells seeded in 96-well format. Treatments were added an hour prior to HCoV-OC43, with the starting concentration of MEO being 1:1000 (0.8469 mg/mL) with a 1:2 serial dilution whereas Remdesvir and Nirmatrelvir starting concentrations were 7 µM and 25 µM, respectively (Figure 3A). After three days post-infection, immunostaining was performed to determine the MEO dose–response curve similar to HCoV-229E assay. The dose–response curve of MEO showed an EC_50_ = 1.405 mg/mL when infected with 1:20 dilution of HCoV-OC43, demonstrating antiviral activity (Figure 3B).

**Figure 4:**
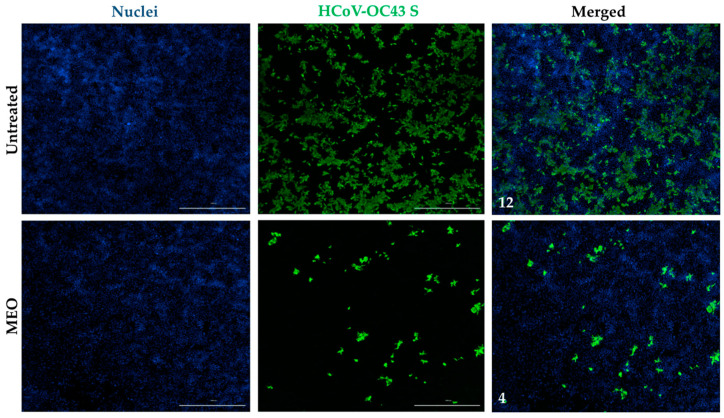
Immunofluorescent staining for HCoV-OC43 spike protein. Huh7.5 cells without MEO treatment showed a mean fluorescence intensity of 12 with HCoV-OC43. At a 1:1000 (0.8469 mg/mL) MEO concentration, HCoV-OC43 viral infection has a mean fluorescence intensity of 4. Nuclei were stained with Hoechst and HCoV-OC43 was stained with anti-coronavirus antibody, OC-43 strain, clone 541-8F, and Alexa Fluor 488, labeled goat anti-mouse secondary antibody, and was imaged at a 1000 µm scale.

Images from the immunostaining assay were captured using the Cytation 7 to validate MEO potency against HCoV-OC43. A 1:1000 (0.8469 mg/mL) MEO dilution image was used to compare the percentage of infection with the untreated well. The MEO-treated well has lower HCoV-OC43 infection in comparison to the untreated well when both are infected with 1:20 HCoV-OC43 (Figure 4). A low dose of MEO is able to inhibit HCoV-OC43 with minimal viable cell death, which indicates that MEO is effective for both HCoV-229E and HCoV-OC43. Moreover, MFI was also used on the HCoV-OC43 of MEO and untreated wells, which shows an MFI of 12 in the untreated well compared to an MFI of 4 in the 1:1000 (0.8469 mg/mL) MEO-treated well (Figure 4). Thus, this supports our dose–response curve finding that HCoV-OC43 infection is inhibited based on the images shown by MEO.

### 2.6. Comparison of EC_50_ and CC_50_ with HCoV-OC43E Infection for MEO, Remdesivir, and Nirmatrelvir

In support of the dose–response curve and MFI, MEO also showed promising inhibition against HCoV-OC43 with an EC_50_ of about 0.017% relative dilution and low cytotoxicity similar to HCoV-229E (Table 4). MEO cytotoxicity is also about three-fold above the EC_50_ for HCoV-OC43, which indicates that MEO exerts antiviral activity without cell death. Remdesivir and Nirmatrelvir were performed in parallel with MEO as control drugs against HCoV-OC43 with EC_50_ of 0.021 µM and 0.012 µM, respectively. Thus, Remdesivir and Nirmatrelvir also exhibited low cytotoxicity and potent inhibition against HCoV-OC43.

### 2.7. Effect of MEO and Remdesivir on SARS-CoV-2 Subgenomic Replicon System

To assess potential antiviral activity of MEO on SARS-CoV-2 coronavirus infection, we used two different SARS-CoV-2 molecular tools, subgenomic replicon systems, and virus-like particle (VLP) systems to identify impacts on viral replication, viral entry, and viral assembly/egress.

For the SARS-CoV-2 subgenomic replicon system we used recapitulates viral replication of SARS-CoV-2 in vitro de-coupled from other stages of the viral life cycle. This system uses an artificial bacterial chromosome plasmid that expresses SARS-CoV-2 replicase complex genes and two reporters, mNeoGreen fluorescent protein (GFP) and Nano-Glo luciferase (NLuc), as previously described [29]. The replicon system bypasses entry through co-transfection of the replicon and a SARS-CoV-2 nucleocapsid plasmid to allow for viral replication and expression of reporter genes, but no generation of new viral particles. Using this system, we can detect if MEO treatment has a direct impact on viral replication using GFP+ cells as a measure of successfully transfected cells that have established viral replication and the NLuc activity in cell culture supernatants to quantify overall viral replication levels in the experiment.

**Figure 5:**
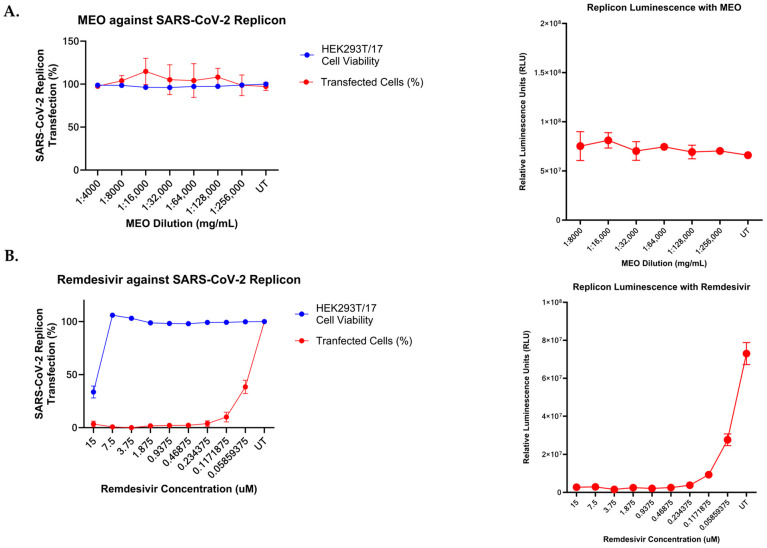
MEO does not inhibit SARS-CoV-2 viral replication based on SARS-CoV-2 replicon. (**A**) Normalized SARS-CoV-2 replicon transfection to determine if MEO inhibits viral replication. Using GFP reporter in the SARS-CoV-2 replicon to calculate the percent of transfected cells, there was no difference in the number of transfected cells compared to normalized and untreated wells. NLuc activity from the SARS-CoV-2 replicon plasmid was used to quantify the amount of viral replication under MEO-treated conditions. Titration of MEO with SARS-CoV-2 replicon transfected cells showed no difference in viral replication via NLuc activity. Cell viability is not affected by SARS-CoV-2 replicon, nor by MEO cytotoxicity at the tested MEO concentrations. The relative luminescence graph validates that viral replicon is not inhibited by MEO. (**B**) Remdesivir substantially inhibited SARS-CoV-2 viral replication and normalized transfection level similar to the relative luminescence units, as untreated wells received about an eight-fold increase compared to 7.5 µM. At 15 µM of Remdesivir, cell viability dropped below 50%, exhibiting Remdesivir cytotoxicity.

To this end, HEK293T/17 cells were co-transfected with the SARS-CoV-2 WA1 replicon and N plasmids, as described in methods Section 2.8. One day post-transfection, the cells were detached and reseeded in a 96-well plate and incubated at 37 °C for one hour before treatment with titrations of either MEO or Remdesivir control drugs. The starting concentration of each drug was a 1:4000 dilution and 15 µM in DMEM (10% FBS + 1% NEAA), respectively, followed by a 1:2 serial dilution. Low concentrations of MEO were used for this assay, as MEO has high cytotoxicity in HEK293T/17 cells that impact the transfection efficiency of cells and is a confounding variable when using the replicon system. We found that pre-treatment of HEK293T/17 cells with MEO at concentrations above 1:5000 (0.16938 mg/mL) followed by transfection with 1 µg of an RFP-expressing control plasmid resulted in reduced transfection efficiency resulting from drug cytotoxicity. RFP transfection efficiency was significantly reduced in cells treated with high MEO concentrations (Supplementary Figure S1A). At higher concentrations of MEO, cell count and RFP+ cells were highly correlated (r^2^ = 0.8838, *p* < 0.0001), indicating that the reduction in transfection efficiency can be attributed to MEO cytotoxicity (Supplementary Figure S1B). The number of GFP+ cells and levels of NLuc activity were measured at four days post-transfection. The data show that the number of GFP+ cells did not change with any concentration of MEO tested compared to untreated control cells, and the NLuc activity in the cell culture supernatant was not altered with MEO treatment, indicating that MEO does not have an antiviral effect on SARS-CoV-2 replication (Figure 5A). Conversely, Remdesivir treatment of replicon-transfected cells shows that replication is significantly impaired, confirming that the replicon assay is specific for effects on viral replication in our model (Figure 5B).

### 2.8. SARS-CoV-2 Virus-like Particles System on MEO and Remdesivir

To assess the effects of MEO on other stages of the SARS-CoV-2 life cycle, we used a virus-like particle approach by encapsidating the SARS-CoV-2 subgenomic replicon plasmid into single-infection particles. This was performed by co-transfecting the SARS-CoV-2 Omicron BA.1 replicon plasmid with the SARS-CoV-2 BA.1 spike protein and nucleocapsid protein expression plasmids as described above.

**Figure 6:**
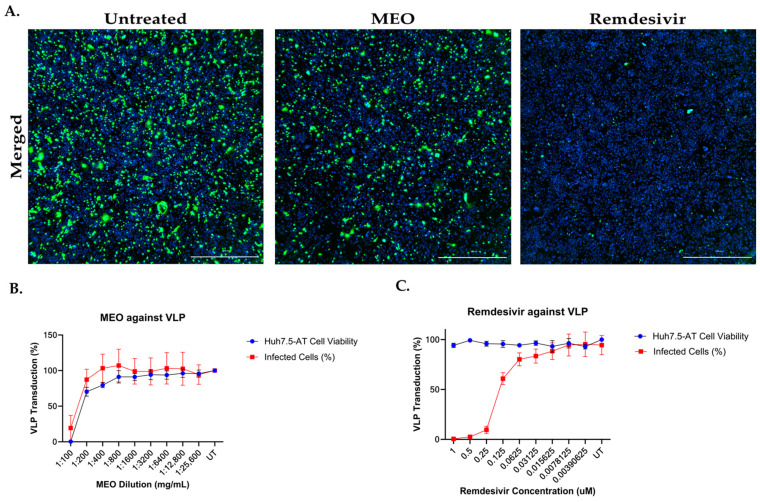
No inhibition against SARS-CoV-2 VLPs for MEO. (**A**) Immunofluorescence images showed no difference in VLP inhibition for untreated and MEO-treated wells at 1:200 (4.2345 mg/mL) concentration. Remdesivir inhibited SARS-CoV-2 VLP in a dose-dependent manner, which acted as positive control. To determine the percentage of cells infected with SARS-CoV-2 VLPs, the cells were counterstained with Hoechst and imaged for GFP reporter signal from the replicon plasmids and Hoechst dye using Cytation 7. All images are in 1000 µm scale. (**B**) The VLP transduction assay with MEO treatment showed similar infection levels as untreated wells. At 1:100 (8.469 mg/mL) concentration of MEO, cells were not viable, and viral replication remained consistent. (**C**) Remdesivir showed potent inhibition against SARS-CoV-2 VLP as an inhibitor of SARS-CoV-2 viral replication.

We first measured the effect of MEO on VLP entry by pre-treating Huh7.5-AT cells with MEO or remdesivir control at a starting concentration of 1:100 (8.469 mg/mL) and 1 µM in DMEM (10% FBS + 1% NEAA), respectively. Each drug was titrated using a 1:2 serial dilution. After one hour, the cells were transduced with the SARS-CoV-2 Omicron BA.1 VLPs. At one day post-transduction, the cell culture supernatant was measured for NLuc activity, and cells were fixed for staining and imaging of GFP+ cells. Imaging of GFP+ cells and the total cell count revealed that there was no effect on the number of cells infected by the VLPs from MEO treatment compared to the untreated VLPs, but that remdesivir-control-treated conditions had significantly fewer GFP+ cells than untreated controls (Figure 6A,B). Titration of MEO treatment also had no effect on NLuc activity in the supernatant of VLP-transduced cells, but remdesivir controls significantly reduced supernatant NLuc activity in a dose-dependent manner (Figure 6C).

**Figure 7:**
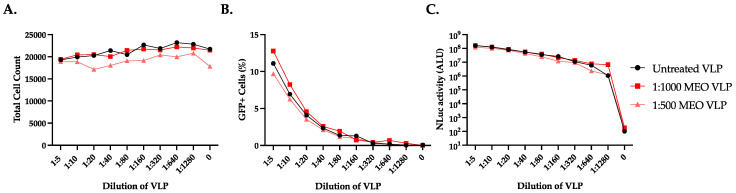
MEO treatment has little impact SARS-CoV-2 Omicron BA.1 VLP formation/release. SARS-CoV-2 BA.1 VLPs were produced in HEK293T/17 cells that were pre-treated with either a 1:1000 (0.8469 mg/mL) or 1:500 (1.6938 mg/mL) concentration of MEO in DMEM (10% FBS + 1% NEAA) to assess the effect of MEO on the formation of infectious VLPs. VLPs were harvested via centrifugation and concentrated 20× using 100,000 MW Amicon filter units. VLPs were titrated on Huh7.5-AT cells at a starting dilution of 1:5 and continued with a 1:2 dilution. At one day post-transduction, the cells were counterstained with 1:5000 Hoechst dye and imaged using Cytation 7 for the number of GFP+ and total cells. Then, the cell culture supernatant was measured for NLuc activity. (**A**) Hoechst staining shows that there was a slight decrease in cell viability for VLPs formed in the presence of MEO treatment in a dose-dependent manner (*p* < 0.0001). (**B**) Analysis of the GFP+ cells representing cells successfully transduced with VLP and undergoing replication of the replicon plasmid showed minor differences between MEO-treated and untreated VLPs, but only at the highest dilutions of VLP delivery (*p* = 0.01). (**C**) NLuc activity shows no difference between MEO VLPs and untreated VLPs. Overall, MEO treatment during VLP production has no effect on nascent VLP particles.

We next measured the effect of MEO on VLP assembly/release by generating VLPs in the presence of MEO treatment. To do this, we seeded HEK293T/17 cells in two 10 cm dishes as described above. After 24 h, we pretreated the cells with either a 1:500 (1.6938 mg/mL) or 1:1000 (0.8469 mg/mL) concentration of MEO in DMEM (10% FBS + 1% NEAA) or no treatment as a control. One hour after treatment, we transfected the cells with the plasmids to generate SARS-CoV-2 Omicron BA.1 VLPs, as described above. VLPs were harvested by collecting the cell culture supernatant from each treatment and centrifuging them for 2 min at 1000× *g* and 4 °C to clarify the supernatant. The clarified supernatant was concentrated 20× using 100,000 NMWL Amicons and centrifuged for 15 min at 1000× *g* and 4 °C. To test the MEO vs. untreated VLPs, we titrated each batch of VLPs on Huh7.5-AT cells seeded in a 96-well format. The starting concentration of VLPs on the Huh7.5-AT cells was 1:5 and the VLPs were serially diluted at a 1:2 rate. VLPs were diluted in DMEM (10% FBS + 1% NEAA). VLPs were applied in 50 µL/well and spinoculated for 1 h, 37 °C, 1000× *g* in the centrifuge. At one day post-VLP transduction, the supernatant was analyzed for NLuc activity, and the cells were stained for total cell count and imaged for GFP+ cells and total cell count. Total cell counts showed that the treatment of cells with MEO VLPs or untreated control VLPs resulted in a slight dose-dependent decrease in cell viability (Figure 7A). According to the number of GFP+ cells, there were minor differences in the percentage of cells successfully transduced with MEO VLPs compared with untreated control VLPs only at the highest concentrations (Figure 7B). This is presumably related to the overall cell count. NLuc activity also revealed that MEO VLPs had no differences in the total amount of replication compared to untreated control VLPs (Figure 7C). Based on these data, MEO treatment may have either an indirect effect or no effect on the SARS-CoV-2 life cycle.

## 3. Discussion

The SARS-CoV-2 pandemic has unveiled the importance of having antiviral drugs available to prevent casualties, with more than 10% of the United States population continuing to be infected as of 22 July 2024 [30]. The consequences of the pandemic have pushed back almost a decade of progress in improving life expectancy, and have exacerbated health inequities in various countries [31]. Moreover, vaccines are prophylactic and do not resolve infections already present in humans. Currently, Remdesivir and Nirmatrelvir are two widely used drugs that are used to treat patients with severe COVID-19. Although Remdesivir is effective in hospitalized patients who are at risk of progressing to severe COVID-19, Remdesivir, a polymerase inhibitor, was deemed to be ineffective against hospitalized patients who are ventilated or at severe risk of death [32,33]. Nirmatrelvir, a protease inhibitor, appears to have a substantial effect on preventing the rates of hospitalization and death from COVID-19 among patients who are over 65 years old, but the drug exhibited minimal evidence in younger adults, leading to a lack of inclusivity from the two prominent treatments available currently in the United States [34]. A combination of synthetic drugs and essential oils allows us to expand our drug remedies to alleviate human coronavirus infections by utilizing natural compounds. Despite the side effects of both Remdesivir and Nirmatrelvir, they are currently the approved treatments for combatting coronaviruses and should not be overlooked, as they are effective treatments for a majority of COVID-19 patients.

In mild coronavirus infections, a combination of synthetic drugs and essential oils allows us to expand our drug remedies to alleviate human coronavirus infections by utilizing natural compounds. In some cases, synthetic drugs may have increased side effects on various organs, while natural products can compensate for the limitations of synthetic drugs by utilizing natural bioactive compounds to inhibit viral infections [35]. According to the previous literature, essential oils with prospective anti-COVID-19 activity show more rapid diffusion through the respiratory system than synthetic drugs and hinder ACE2 receptor-binding to SARS-CoV-2 spike proteins in the lung parenchyma [36]. The study of essential oils in another human coronavirus like HCoV-229E may reveal novel broad-spectrum antivirals to combat SARS-CoV-2 [37,38]. Even though our study does not indicate MEO to be highly potent in treating HCoV-229E and HCoV-OC43 in comparison to our control drugs, MEO can be beneficial in mild infections and decrease the side effects that synthetic drugs may have, although synthetic drugs should be the treatment option in severe cases. MEO still exhibited a decrease in infected cells by about 70% in HCoV-229E infection with 1:2000 (0.42345 mg/mL) and 1:1000 (0.8469 mg/mL) concentrations in HCoV-OC43 infected cells.

Although little information about the MEO is known, a previous study showed antimicrobial and antifungal activities on food spoilage pathogens. The study demonstrated that MEO exhibited higher antibacterial activity on Gram-positive bacteria than on Gram-negative bacteria using minimum inhibitory concentrations (MICs) [39]. Furthermore, MEO was able to inhibit *Fusarium* sp. and *Aspergillus* sp., which are known for food spoilage, using a minimum fungicidal concentrations assay [39]. Thus, with our paper demonstrating antiviral activity against human coronaviruses, MEO appears to have potential effects on fungal, viral, and bacterial inhibitions, and may be an effective essential oil for other species.

In this paper, we have revealed that MEO is able to successfully inhibit HCoV-229E and HCoV-OC43 viruses, but MEO showed no antiviral activity against SARS-CoV-2 using subgenomic replicon and VLP systems. By using these novel systems for SARS-CoV-2, we can determine if MEO inhibits viral replication and viral assembly/egress based on transfection and viral entry by transducing Huh7.5-AT with VLPs. This approach enabled us to target a specific pathway that MEO could inhibit in SARS-CoV-2. However, MEO showed no signs of inhibition across various viral stages, unlike Remdesivir, which significantly inhibited viral replication. HEK293T/17 cells were used for SARS-CoV-2 studies, since these cells are easily transfected with our bacterial artificial chromosomes for replicon while Huh7.5-AT allowed us to compare the results with HCoV-229E and HCoV-OC43 infections. Although the exact mechanisms that inhibit HCoV-229E and HCoV-OC43 infections are yet to be determined, MEO’s slight level of antioxidant activity may play a role in its antiviral potency. The previous literature has shown that antiviral drugs with high antioxidant activity protect cells against oxidative damage in the mitochondrial respiratory chain and oxidative stress by influenza virus [40].

This study was not conducted without limitations. First, the extraction of the plant-derived *Myrtus communis* L. essential oil may not be grown at an optimal condition such as the controlled humidity, temperature, or soil nutrients in the environment. Second, we do not know the expiration date and time frame for the essential oil extract to remain effective. To control this, we tested the essential oil within 6 months of receiving the essential oils, and we repeated this with two different batches. However, we were able to see a decrease in antiviral effects after experimenting with the MEO on Huh7.5 cells after 6 months of usage. Third, the HCoV-229E and HCoV-OC43 infections were performed on hepatoma cell line Huh7.5 cells instead of more relevant lung cell lines like A549 or Calu3. Fourth, while the SARS-CoV-2 subgenomic replicon system allowed us to target specific viral pathways, utilizing live SARS-CoV-2 would be beneficial in validating our findings. Lastly, the composition of essential oils is not created based on the exact compositions of the pure compounds; thus, the mechanistic pathways of various chemical properties in MEO can alter the antiviral effectiveness and variable potency because of biological variability.

The current study helps validate that MEO contains antiviral activity against HCoV-229E and HCoV-OC43 and can advance further studies regarding the exact pathways and specific chemical compounds that decrease HCoV-229E and HCoV-OC43 infections along with other coronaviruses. Our study here elucidated that *Myrtus communis* L. essential oil demonstrates potential antiviral activity against HCoV-229E and HCoV-OC43 in Huh7.5 cells within a low cytotoxicity range. This preliminary study shines a light on MEO as an alternative natural remedy to combat human coronaviruses and may possess a potential antiviral strategy to tackle other human coronaviruses. Future investigations on testing specific chemical compositions within the MEO will allow us to analyze similarities across other essential oils and allow us to pinpoint specific molecules that demonstrate antiviral effects on coronaviruses. Lastly, this study provides value to the natural products library and offers possible therapeutics for various infectious diseases.

## 4. Materials and Methods

### 4.1. Myrtus Essential Oil Extraction

Aerial parts, particularly the leaves, were collected by Dr. Lucia Cascioni (owner of the herbal company “Erbe di Sardegna”) in April 2021. Tempio Pausania (40°54′05.33″ N 9°05′15.7″ E) is a municipality in the Province of Sassari in the historical Gallura subregion in the island of Sardinia in Italy. Representative plant specimens have been deposited at the Skin Lab (cumulative identification number: 16733/EO) of the Department of Biomedical Sciences of the University of Sassari. A density of 0.8469 g/mL was obtained from the MEO extract.

### 4.2. Gas Chromatography—Mass Spectrometry (GC-MS) Analysis

This study was conducted using an advanced Agilent 7890 GC system equipped with a Gerstel MPS autosampler. This system was further integrated with an Agilent 7000C MSD detector (Agilent Technologies, Santa Clara, CA, USA) to enhance detection capabilities. The chromatographic separation process was meticulously performed using two distinct columns: a VF-Wax column, measuring 60 m in length and 0.25 mm in internal diameter, with a film thickness of 0.5 μm, and a capillary column HP-5MS, which was 30 m long and 0.25 mm in diameter, with a thinner film thickness of 0.17 μm. Both columns are products of Agilent Technologies from Santa, Clara, CA, USA.

A specific temperature program was used for the VF-Wax column as follows: starting from 40 °C, this temperature was held for 4 min, followed by a gradual increase up to 150 °C at a rate of 5.0 °C/min, then maintained at this level for 3 min. Subsequently, the temperature was increased to 240 °C at a rate greater than 10 °C/min and held for another 12 min. In contrast, the HP-5MS column followed a different temperature regime, starting at 60 °C for 3 min, then the temperature was raised to 210 °C at a rate of 4 °C/min and held stable for 15 min. This was followed by an increase to 300 °C at a rate of 10 °C/min, where it remained for an additional 15 min.

Helium was the carrier gas of choice for both columns, flowing at a constant rate of 1 mL/min. The collected data were meticulously analyzed using MassHunter Workstation B.06.00 SP1 software. The identification of the individual components, as detailed in Table 1, was achieved through a global approach which involved comparison with co-injected pure compounds. This process also included matching MS fragmentation patterns and retention indices to established references, such as built-in libraries or literature data and commercial mass spectral libraries, specifically purchased from NIST/EPA/NIH 2008 and HP1607 by Agilent Technologies.

### 4.3. Antiradical Activity of Essential Oil from Myrtus

The antiradical activity of MEO was measured using the 2,2-diphenyl-1-picrylhydrazyl (DPPH) assay, modified from the method of a previous study [41]. Various EO concentrations, ranging from 0.1 to 2 mg, were mixed with DPPH (100 µM in ethyl acetate) to a total volume of 1 mL. These mixtures were incubated at 25 °C in the dark. The DPPH radical reduction was gauged by recording the absorbance at 517 nm at specific intervals (60, 180, and 300 min) until stabilization and using an Ultrospec 4300 pro UV–Vis spectrophotometer (Amersham Biosciences, Freiburg, Germany). A Trolox calibration curve (0.25–7.5 µg/mL) served as the standard for quantifying DPPH radical activity.

The scavenging activity of the DPPH radical was calculated as follows:

% scavenging of DPPH radical = [(A_blank_ − A_sample_)/A_blank_] × 100

where A_blank_ is the absorbance of the control reaction (containing all reagents except for the test compound), and A_sample_ is the absorbance in the presence of MEO. Experiments were carried out in triplicate, and results were expressed as mean ± SD.

### 4.4. Cell Culture

Human hepatoma-derived cells (Huh7.5) were obtained from Dr. Charles M Rice’s Laboratory (The Rockefeller University, NY). Human embryonic kidney cells with epithelial morphology (HEK293T/17) and Huh7.5 with overexpressed ACE2 and TMPRSS2 (Huh7.5-AT) were obtained from Dr. Stefan Sarafianos’ lab (Emory University School of Medicine, Atlanta, GA, USA). The cells were cultured at 37 °C with 5% CO_2_ humidified incubator in Dulbecco’s Modified Eagle’s Medium (Fisher Scientific, Waltham, MA, USA) with 10% Fetal Bovine Serum (FBS) and 1% Non-Essential Amino Acids (NEAA).

### 4.5. HCoV-229E and HCoV-OC43 Infection and Inhibition Assays

Huh7.5 cells were seeded on collagen-coated 96-well tissue culture plates (Falcon, Corning, NY, USA. Cells were treated with a 1:2 serial dilution of Myrtus essential oil, Remdesivir (#30354; Cayman Chemicals, MI, USA), or Nirmatrelvir (PF-07321332; MedKoo Biosciences, Durham, NC, USA) 24 h after seeding. The starting concentration of Myrtus was 1:1000 relative dilution in Dulbecco’s Modified Eagle’s Medium (DMEM) with 10% FBS and 1% NEAA, and the starting concentrations for Remdesivir and Nirmatrelvir were 250 nM and 25 µM, respectively. One hour after treatment, HCoV-229E virus or HCoV-OC43 diluted in DMEM (10% FBS and 1% NEAA) was added on top of the drug treatments. Cells were fixed with 4% paraformaldehyde (PFA) at one day post-infection for HCoV-229E and three days post-infection for the HCoV-OC43 assay.

### 4.6. HCoV-229E and HCoV-OC43 Immunofluorescence Staining and Imaging

Cells were permeabilized with 1:1000 Triton-X in PBS for 10 min at room temperature and washed with PBS. After washing, 5% goat serum in PBS (Jackson ImmunoResearch, West Grove, PA, USA) was used for blocking for at least one hour. HCoV-229E infected cells were stained for HCoV-229E Spike RBD (#MAB10938; Bio-Techne, Minneapolis, MN, USA) in 5% goat serum solution overnight at 4 °C, whereas HCoV-OC43-infected cells were stained for Anti-Coronavirus Antibody, OC-43 strains, and clone 541-8F (#MAB9012; Sigma-Aldrich, St. Louis, MO, USA). Cells were washed with 0.1% Tween-20 (H5151, Promega, Madison, WI, USA) in PBS, and AlexaFluor 488 Goat anti-mouse IgG (H+L) Cross-Absorbed Secondary Antibody (#AA11001; Thermo Fisher Scientific, Waltham, MA, USA) was added in a 1:1000 relative dilution. Cells were counterstained with 1:1000 Hoescht (#33342; Thermo Fisher Scientific) with 0.1% Tween-20 in PBS for an hour in the dark. Cells were imaged using the Cytation 7 Cell Imaging Multimode Reader (BioTek, Agilent, Santa Clara, CA, USA). Cell viability and infected cell data were obtained, and a dose–response curve was generated using Prism 10 to identify EC_50_ values.

Images of infected cells were also captured using Cytation 7 Imaging Multimode Reader. Specifically, merged images of both nuclei and HCoV-229E spike protein from infected/untreated wells were also separated to show only nuclei or only HCoV-229E spike protein images. At 1:2000 (0.42345 mg/mL) concentration of MEO, merged images of the nuclei and HCoV-229E spike protein were compared to the merged untreated image. A similar method was used for HCoV-OC43, but a 1:1000 (0.8469 mg/mL) concentration of MEO was used to compare with the merged untreated image. ImageJ software (version 1.54c) was used to measure the mean fluorescence intensity.

### 4.7. PrestoBlue Cell Viability Assay for Huh7.5

Huh7.5 cells were seeded in 96-well collagen-coated plates in DMEM (10% FBS and 1% NEAA) for Remdesivir, Nirmatrelvir, and MEO treatment. After confluency, a 1:2 serial dilution with triplicates for each drug was used. A 1:40 (21.1725 mg/mL) concentration was used as the starting concentration for MEO. An amount of 1000 nM and 100 µM relative starting dilutions were used for Remdesivir and Nirmatrelvir, respectively. One hour after drug treatment, DMEM (10% FBS and 1% NEAA) was added on top of the drug media to mimic the addition of HCoV-229E and HCoV-OC43 viruses. One day post-infection, PrestoBlue Cell Viability Reagent (#A13261; Thermo Fischer) was mixed with DMEM (10% FBS and 1% NEAA) using a 1:10 ratio and filtered through a 0.45 µm filter. Media was removed and PrestoBlue was added to each well. The plate was incubated at 37 °C in a 5% CO_2_ humidified incubator for an hour. The media was transferred to 96-well black cell culture plates with clear bottoms and was imaged within 7 h using a BioTek Cytation 7 Cell Imaging Multimode Reader in a 1000 µm scale to detect DAPI. The number of cells was obtained, and a PrestoBlue cell viability curve was analyzed using Prism 10. The CC_50_ values for Remdesivir, Nirmatrelvir, and MEO were obtained to create a table.

### 4.8. SARS-CoV-2 MEO Replicon Assay and Luminescence

HEK293T/17 cells were seeded in a 10 cm dish in DMEM (10% FBS and 1% NEAA). Half of the media was removed and replenished with DMEM (10% DMEM and 1% NEAA) in the morning prior to transfection. During transfection, 500 µL of jetPRIME buffer and 12 µL of jetPRIME transfection (#55-132, Polyplus-transfection, Illkirch, France) were mixed with SARS-CoV-2 Washington strain plasmids (WA1 plasmids #304 (nucleocapsid) and #518 (subgenomic replicon)) obtained from Dr. Stefan Sarafianos’ lab (Emory University School of Medicine, Georgia) [29]. A ratio of 1 µg and 5 µg of the plasmids, respectively, were used for the transfection reagent and were incubated for 20 min at room temperature before being applied to the cells in a dropwise manner. One day post-transfection, the cells were trypsinized with trypsin-EDTA 0.05%, phenol red (#25300054, Gibco, Thermo Disher Scientific, Waltham, MA, USA) for 2 min. Detached cells were reseeded in a collagen-coated 96-well tissue culture plate (Falcon) and incubated for an hour. A starting concentration of 1:4000 (0.211725 mg/mL) and 15 µM of MEO and Remdesivir were added after an hour with a 1:2 serial dilution, respectively. Four days post-transfection, the cells were live stained with 1:1000 Hoechst (#33342; Thermo Fisher Scientific) in DMEM (10% FBS and 1% NEAA), and the supernatant was collected for luminescence analysis in a 96-well white-bottom plate (Falcon). A 1:50 dilution of Nano-Glo luciferase substrate in the provided luciferase buffer (#N1120, Promega) was added on top of the supernatant, and relative luminescence units were obtained using a GloMax Navigator Microplate Luminometer (Promega, Madison, WI, USA) for MEO and Remdesivir.

### 4.9. MEO Transfection Efficiency Assay

In total, 850,000 HEK293T/17 cells were seeded in a 6-well plate in 2 mL/well of DMEM (10% FBS and 1% NEAA). The following day, cells were pre-treated with either a 1:500 (1.6938 mg/mL), 1:1000 (0.8469 mg/mL), 1:5000 (0.16938 mg/mL), or 1:10,000 (0.08469 mg/mL) dilution of MEO or matched DMSO control by removing 1 mL of media from each well of cells and applying 1 mL of fresh medium with twice the final desired drug concentration. One hour after treatment, cells were transfected with 1 µg/well of pSCRPSY-Empty plasmid (transfection control) using 4.4 µL/well of jetPRIME transfection reagent (#55-132, Polyplus-transfection) and 84.75 µL/well of the matching jetPRIME transfection buffer, added dropwise. Cells were incubated overnight at 37 °C. At 24 h post-transfection, 1 mL of media was removed from each well. An amount of 1 mL of fresh DMEM (10% FBS + 1% NEAA) containing Hoechst dye (mixed at 2× to result in a final concentration of 1:5000 in each well) was added. Cells were incubated with the dye for 30 min at 37 °C. Another half-media change was performed to remove unbound Hoechst dye, and then the cells were imaged with Cytation 7 to quantify the total number of cells and positive red fluorescent protein (RFP+) reporter expression from the pSCRPSY-Empty plasmid.

### 4.10. SARS-CoV-2 Virus-like Particles Production and Titration

In a 10 cm dish (#CLS430165, Corning, Corning, NY, USA), 5 million HEK293T/17 cells were seeded 24 h before transfection. In the morning prior to transfection, 5 mL of DMEM (10% DMEM and 1% NEAA) was removed and replenished with 6 mL of DMEM (10% DMEM and 1% NEAA). At 24 h after the seeding mark, 500 µL of jetPRIME buffer and 20 µL of jetPRIME transfection reagent (#55-132, Polyplus-transfection) were mixed with SARS-CoV-2 Omicron BA.1 plasmids #535 (nucleocapsid), #538 (spike protein), and #603 (replicon) obtained from Dr. Stefan Sarafianos’ lab (Emory University School of Medicine, Georgia) [29]. A ratio of 1 µg, 1 µg, and 5 µg of the plasmids were used, respectively, in the reagent and incubated for 20 min at room temperature. The reagent was added on top of the media in the 10 cm dish in a dropwise manner. One day post-transfection, 6 mL of the media was removed and replenished with 7 mL of DMEM (10% DMEM and 1% NEAA). After 4 days post-transfection, the supernatant was centrifuged for 5 min at 1500× *g* and recollected the supernatant to concentrate the virus-like particles (VLPs). VLPs were concentrated using a 100,000 NMWL Amicon (#UFC910008, Millipore Sigma, Burlington, MA, USA) and were centrifuged for 20 min at 1500× *g*. The concentrated VLPs were stored at −80 °C prior to the VLP titration assay.

Huh7.5-AT cells were seeded in a collagen-coated 96-well tissue culture plate (Falcon) a day before VLP transduction. A starting concentration of 3:4 of VLP with a 1:2 serial dilution in DMEM (10% FBS and 1% NEAA) was used to determine the optimal dilution for transduction. After transduction, cells were spinoculated in the centrifuge at 1000× *g* 37 °C for an hour. Cells were fixed with 4% paraformaldehyde (PFA) at one day post-transduction and stained with 1:1000 Hoechst (#33342; Thermo Fisher Scientific) in PBS for an hour and washed three times prior to imaging using Cytation 7.

### 4.11. SARS-CoV-2 Virus-like Particles Transduction Assay and Immunofluorescence Staining

A collagen-coated 96-well tissue culture plate (Falcon) was used to seed Huh7.5-AT cells. At the 24 h mark, MEO and Remdesivir were added at a starting concentration of 1:100 (8.469 mg/mL) and 1 µM using a 1:2 serial dilution, respectively. An hour after drug treatment, 3:64 of VLPs were added on top and spinoculated in the centrifuge for an hour at 37 °C and 1000× *g*. Cells were fixed with 4% paraformaldehyde (PFA) at one day post-transduction and washed with PBS. A 1:1000 dilution of Hoechst (#33342; Thermo Fisher Scientific) was mixed with PBS for staining. After an hour of Hoechst staining, 3 washes were performed prior to imaging. Transduction inhibition, cell viability, images, and transduction percentages were extracted from Cytation 7 for analysis.

## Figures and Tables

**Table 1:** Chemical composition of *Myrtus communis* L. essential oil (MEO). Data are the mean of three replicates ± SD. Identification methods (IM): MS by comparison of the Mass spectrum with those of the computer mass libraries Adams, Nist 11, and by interpretation of the mass spectra fragmentations. RI by comparison of retention index with those reported in the literature. Std by comparison of the retention time and mass spectrum of available authentic standards. RT (retention time), KI let (Kovats retention index), let (literature), and sper (experimental).

Rt	KI Let	KI Sper	Components	% ± SD	IM
12.242	892	892	isobutyl isobutyrate	0.25 ± 0.02	MS-RI
12.626	930	929	artemisia triene	0.10 ± 0.01	MS-RI
12.781	939	939	endo-5-norbornene-2-ol	0.12 ± 0.01	MS-RI
13.068	939	940	α-pinene	37.83 ± 0.15	Std
13.552	944	947	5-methyl-3-heptanone	0.06 ± 0.02	MS-RI
13.617	954	956	camphene	0.60 ± 0.03	Std
14.492	979	977	*trans*-para-menthane	0.21 ± 0.02	MS-RI
14.640	979	979	ß-pinene	0.38 ± 0.02	Std
15.047	986	985	*cis*-pinane	0.20 ± 0.01	MS-RI
16.281	1025	1025	*p*-cymene	1.59 ± 0.05	Std
16.450	1029	1030	limonene	10.70 ± 0.09	Std
16.562	1031	1032	1,8-cineole	27.17 ± 0.8	Std
17.463	1060	1058	γ-terpinene	0.19 ± 0.03	Std
18.759	1097	1097	linalool	7.24 ± 0.10	Std
19.077	1102	1101	*cis*-thujone	0.31 ± 0.04	MS-RI
19.425	1114	1114	*trans*-thujone	0.17 ± 0.02	MS-RI
19.962	1134	1137	1-terpineol	0.17 ± 0.01	Std
20.288	1139	1139	*trans*-ß-dihydro-terpineol	0.40 ± 0.05	MS-RI
20.393	1146	1149	camphor	0.15 ± 0.01	Std
21.368	1177	1172	terpinene-4-ol	0.33 ± 0.02	Std
21.751	1189	1188	α-terpineol	2.84 ± 0.10	MS-RI
21.954	1194	1192	dihydrocarveol	0.94 ± 0.07	MS-RI
23.355	1243	1242	carvone	0.29 ± 0.02	Std
23.529	1267	1263	geranial	2.32 ± 0.07	Std
24.397	1349	1350	terpinyl acetate	0.12 ± 0.01	MS-RI
30.077	1495	1493	methyl-isoeugenol	1.18 ± 0.04	MS-RI
			Total	95.77	

**Table 2:** Percent scavenging of DPPH radical of MEO. Data were collected at three time points (60, 180, 300 min) for each concentration in triplicate by MEO. Data are expressed as mean ± SD of three independent assays.

mg/mL—Time Points	MEO
2 (60 min)	36.2 ± 9.3
1 (60 min)	28.7 ± 5.0
0.5 (60 min)	18.9 ± 3.6
0.2 (60 min)	1.3 ± 0.7
0.1 (60 min)	0.4 ± 0.8
2 (180 min)	39.1 ± 9.9
1 (180 min)	33.0 ± 8.6
0.5 (180 min)	24.1 ± 9.4
0.2 (180 min)	1.3 ± 2.3
0.1 (180 min)	0.2 ± 0.9
2 (300 min)	40.7 ± 8.8
1 (300 min)	34.1 ± 8.7
0.5 (300 min)	26.3 ± 1.6
0.2 (300 min)	1.4 ± 0.6
0.1 (300 min)	1.8 ± 0.4

**Table 3:** EC_50_ and CC_50_ of HCoV-229E antivirals. A dose–response curve was used to determine EC_50_ and CC_50_ in Huh7.5 cells. Remdesivir and Nirmatrelvir were used as the control antivirals against HCoV-229E.

Compound	EC_50_ (nM)	CC_50_ (nM)
Remdesivir	0.003	9.662
Nirmatrelvir	1.243	337.9
Compound	EC_50_ (mg/mL)	CC_50_ (mg/mL)
MEO	0.1204	4.197

**Table 4:** EC_50_ and CC_50_ of HCoV-OC43 antivirals. A dose–response curve was used to determine EC_50_ and CC_50_ in Huh7.5 cells. Remdesivir and Nirmatrelvir were used as the control antivirals against HCoV-OC43.

Compound	EC_50_ (uM)	CC_50_ (nM)
Remdesivir	0.021	9.662
Nirmatrelvir	0.012	337.9
Compound	EC_50_ (mg/mL)	CC_50_ (mg/mL)
MEO	1.405	4.197

## Data Availability

The original contributions presented in the study are included in the article/Supplementary Material, further inquiries can be directed to the corresponding author.

## Supplementary Materials

The following supporting information can be downloaded at: https://www.mdpi.com/article/10.3390/ph17091189/s1, Figure S1: MEO pre-treatment on HEK293T/17 cells reduces transfection efficiency via cytotoxicity.
