# Supplementary material for: Myrtus communis L. Essential Oil Exhibits Antiviral Activity against Coronaviruses
Source: Pharmaceuticals (Basel). 2024 Sep 10;17(9):1189. doi: 10.3390/ph17091189 (PMC11435418; doi:10.3390/ph17091189)
Supplement: Supplementary file 1 [file pharmaceuticals-17-01189-s001.zip › pharmaceuticals-3154797-supplementary.pdf]

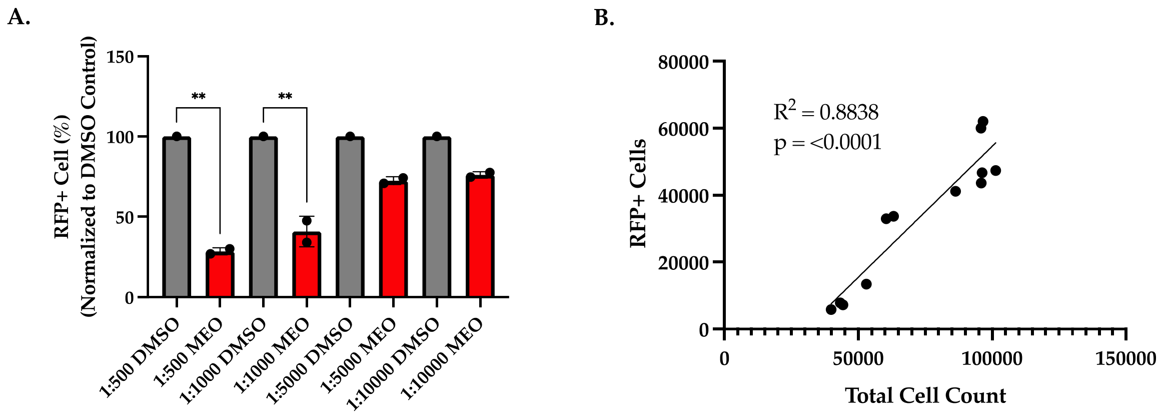

Figure S1. MEO pre-treatment on HEK293T/17 cells reduces transfection efficiency via cytotoxicity. A) HEK293T/17 cells seeded in a 6-well plate were pre-treated with varying concentrations of MEO diluted in DMEM (10% FBS and 1% NEAA) for 1 hour before being transfected with 1 µg of an RFP control plasmid (pSCRPSY-Empty). At 24 hours post-transfection, cells were stained with Hoechst and imaged for RFP-expression and total cell count with the Cytation 7 multimode imager. Results showed that transfection efficiency was significantly reduced in cells pre-treated with MEO above 1:5000 (0.16938 mg/mL). (Ordinary one-way ANOVA with Tukey's test. **p-value < 0.01). B) Simple linear regression analysis of total cell count compared to the number of RFP+ cells from the experiment in panel A shows a significant correlation. (R^2 = 0.8838, p = <0.0001) (1:500 MEO dilution = 1.6938 mg/mL, 1:1000 MEO dilution = 0.8469 mg/mL, and 1:10000 MEO dilution = 0.08469 mg/mL)
